# CAS: corpus of clinical cases in French

**DOI:** 10.1186/s13326-020-00225-x

**Published:** 2020-08-06

**Authors:** Natalia Grabar, Clément Dalloux, Vincent Claveau

**Affiliations:** 1 CNRS, UMR 8163, Lille, F-59000 France; 2 Univ. Lille, UMR 8163 - STL - Savoirs Textes Langage, Lille, F-59000 France; 3 Univ Rennes, Inria, CNRS, IRISA, Rennes, F-35000 France

**Keywords:** Medical area, Natural language processing, Corpus with clinical cases, Morpho-syntactic and semantic annotation, Sustainability, Reproducibility

## Abstract

**Background:**

Textual corpora are extremely important for various NLP applications as they provide information necessary for creating, setting and testing those applications and the corresponding tools. They are also crucial for designing reliable methods and reproducible results. Yet, in some areas, such as the medical area, due to confidentiality or to ethical reasons, it is complicated or even impossible to access representative textual data. We propose the CAS corpus built with clinical cases, such as they are reported in the published scientific literature in French.

**Results:**

Currently, the corpus contains 4,900 clinical cases in French, totaling nearly 1.7M word occurrences. Some clinical cases are associated with discussions. A subset of the whole set of cases is enriched with morpho-syntactic (PoS-tagging, lemmatization) and semantic (the UMLS concepts, negation, uncertainty) annotations. The corpus is being continuously enriched with new clinical cases and annotations. The CAS corpus has been compared with similar clinical narratives. When computed on tokenized and lowercase words, the Jaccard index indicates that the similarity between clinical cases and narratives reaches up to 0.9727.

**Conclusion:**

We assume that the CAS corpus can be effectively exploited for the development and testing of NLP tools and methods. Besides, the corpus will be used in NLP challenges and distributed to the research community.

## Background

Textual corpora are central for various NLP applications as they provide information necessary for creating, setting, testing and validating these applications, the corresponding tools, and the results. Yet, in some areas, due to confidentiality or to ethical reasons, it is complicated or even impossible to access representative textual data typically created and used by the actors of these areas. For instance, medical and legal areas are concerned with these issues: in the legal area, information on lawsuits and trials remains confidential, while in the medical area, medical confidentiality must be respected by the medical staff. In both situations, personal data cannot be made publicly available, which prevents corpora from being released and makes experiments non-reproducible by other researchers and with other methods. To face such situations, Natural Language Processing (NLP) proposes specific methods and tools. Hence, for several years now, anonymization and de-identification methods and tools have been made available and provide competitive and reliable results [1–4] reaching up to 90% precision and recall. But it may still be difficult to access de-identified documents and use them for research. One reason is that there is a risk of re-identification of people, and more particularly of patients [5, 6] because medical histories can be unique. In consequence, the application of de-identification tools on personal data often does not permit to make the data freely available and usable within the research context.

Yet, there is a real need for the development of methods and tools for several applications suited for such restricted areas. For instance, in the medical area, it is important to design suitable tools for information retrieval and extraction, for recruiting patients for clinical trials, for performing several other important tasks such as indexing, study of temporality, negation, etc. [7–13]. Another important issue is related to the reliability of tools and to the reproducibility of study results across similar data from different sources. The scientific research and clinical communities are indeed increasingly coming under criticism for the lack of reproducibility in the biomedical area [14–16], but notice that, for instance, psychology is concerned with this issue as well [17–19]. The first step towards the reproducibility of results is the availability of freely usable tools and corpora. In the current contribution, we are mainly concerned with the construction of freely available corpora for the medical domain. Yet, we are aware that sharing tools and methods is also important. We assume that availability of corpora may boost the design and dissemination of other resources, methods and tools for biomedical tasks and applications.

The purpose of our work is to introduce the CAS corpus, that contains clinical cases in French such as those published in scientific literature or used in the education and training of medical students. In what follows, we first present some existing studies on medical corpora creation (“Existing work: freely available clinical corpora”), highlighting corpora which are freely available for research. We then present the methods used for building, annotation and analysis of the CAS corpus with clinical cases in French (“Methods”). The results are presented in “Results” and discussed in “Discussion”. We conclude with some directions for future work (“Conclusion” sections). The work presented in this article is an extended and updated version of our previous publication [20].

## Existing work: freely available clinical corpora

Within the medical area, we can distinguish two main types of medical corpora: scientific and clinical.

- *Scientific corpora* are issued from scientific publications and reporting. Such corpora are becoming increasingly available to researchers thanks to recent and less recent initiatives dedicated to open publication, such as those promoted by the NLM (National Library of Medicine) through the PUBMED portal^1^ and specifically dedicated to the biomedical area, and by the HAL^2^ and ISTEX^3^ initiatives, which provide generic portals for accessing scientific publications from various areas, including medicine. Such corpora contain scientific publications that describe research studies: motivation, methods, results and issues on precise research questions. Other portals may also provide access to scientific literature aimed at specific purposes, namely indexing reliable literature, such as proposed by HON [21], CISMEF [22], and other similar initiatives [23]. Some existing scientific corpora also provide annotations and categorizations, such as PoS-tagging [24] and negation [25]. These are often built for the purposes of shared tasks [26, 27].
- *Clinical corpora* are related to hospital and clinical events of patients. Such corpora typically contain documents that describe medical history of patients and the medical care they are undergoing. This kind of corpora is typically created and used in clinical context as part of the healthcare process. Even after de-identification, it is complicated to obtain free access to this kind of medical data and, for this reason, there are very few clinical corpora freely available for research.

In our work, we are mainly interested in clinical corpora: the proposed literature review of the existing work is aimed at clinical corpora that are freely available for research. We present here the main existing clinical corpora:

- *MIMIC* (Medical Information Mart for Intensive Care), now available in its third version, provides the largest available set of structured and unstructured clinical data in English. MIMIC III is a single-center database comprising information pertaining to patients admitted in critical care units at a large tertiary care hospital. Those data include vital signs, medications, laboratory measurements, observations and notes charted by care providers, fluid balance, procedure codes, diagnostic codes, imaging reports, hospital length of stay, survival data, and more. The database supports applications including academic and industrial research, quality improvement initiatives, and higher education coursework [28]. Those data are widely used by researchers, for instance for predicting mortality [29, 30], for diagnosis identification and encoding [31, 32], for studies on temporality [33] or for identifying similar clinical notes [34], to cite just a few existing studies. Data from these corpora are also used in challenges, such as i2b2, n2c2 and CLEF-eHEALTH.
- *i2b2* (Informatics for Integrating Biology and the Bedside)^4^ is an NIH-funded initiative promoting the development and test of NLP tools for English-language documents with the purpose of healthcare improvement. In order to enhance the ability of NLP tools to process fine-grained information from clinical records, i2b2 challenges provide sets of fully de-identified clinical notes enriched with specific annotations [9, 11, 35], such as: de-identification, smoking status, medication-related information, semantic relations between entities, or temporality. The clinical corpora and their annotations built for the i2b2 NLP challenges are available now for general research purposes.
- *n2c2* (National NLP Clinical Challenges),^5^ held in 2018 and 2019, also address the processing of English-language clinical documents. These challenges are dedicated to other typical tasks when handling clinical documents: inclusion of patients in clinical trials, detection of adverse-drug events, computing of textual semantic similarity, concept normalization, and extraction of family history.
- *CLEF-eHEALTH* challenges^6^ held in 2013 and 2014 provide annotations for disorder detection and abbreviation normalization. In 2016 the focus was on structuring Australian free-text nurse notes. Finally, in 2016 and 2017 death reports in French, provided by the CépiDc,^7^ have been processed for death cause extraction.
- *eHealth-KD* 2019 challenge^8^ targets human language modelling in a scenario in which electronic health documents in Spanish could be machine readable from a semantic point of view. The two proposed tasks are: identification and classification of key phrases, and detection of semantic relations between these key phrases.

Finally, medical data, close to those handled in the clinical context, can be found in clinical trials protocols. One example is the corpus of clinical trials annotated with information on numerical values in English [36], and on negation in French and Brazilian Portuguese [37, 38].

## Methods

We first describe the specificity of the sources and clinical cases from which the CAS corpus was created (“Building the corpus”), then the annotation rationale (“Annotation of the corpus”), and the principles of its comparison with similar clinical narratives from Rennes University Hospital (“Comparison with clinical narratives” sections).

### Building the corpus

The CAS corpus in French contains clinical cases as published in scientific literature, legal or training material. Hence, it is built using material freely available in online sources. The collected clinical cases are published in different journals and websites from French-speaking countries in various continents. Those clinical cases are related to various medical specialties (e.g. cardiology, urology, oncology, obstetrics, pulmonology, gastro-enterology...).

The purpose of clinical cases is to describe clinical situations for real de-identified or fake patients. Common clinical cases are typically part of education programs used for training medical students, while rare cases are usually shared through scientific publications to illustrate less common clinical situations. As for clinical cases which can be found in legal sources, they usually report on situations which became complicated due to various reasons emanating from different healthcare levels: medical doctor, healthcare team, institution, health system and their interactions.

Similarly to clinical documents, the content of clinical cases depends on the clinical situations that are illustrated, and on the disorders, but also on the purpose of the presented cases: description of diagnoses, treatments or procedures, evolution, family history, adverse-drug reactions, expected audience, etc.

Data in published clinical cases are de-identified by the authors prior to their publication. Besides, publication is usually done with the written permission of patients. The case reports can be related to any medical situation (diagnosis, treatment, procedure, follow-up...), to any specialty and to any disorder. The typical structure of scientific publications with clinical cases starts by introducing the clinical situation, then one or more clinical cases are presented to support the situation. Schemes, imaging, examination results, patient history, lab results, clinical evolution, treatment, etc. can also be provided for the illustration of clinical cases. Finally, those clinical cases are discussed. Hence, such cases may present an extensive description of medical problems. Such publications gather medical information related to clinical discourse (clinical cases) and to scientific discourse (introduction and discussion). The related scientific references are also provided.

**Fig. 1:**
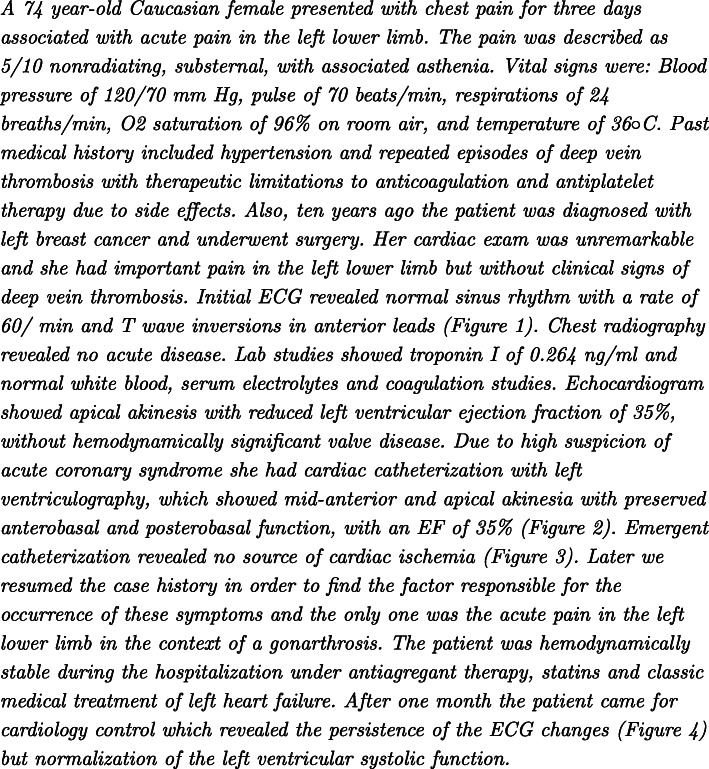
Example of clinical case

Figure 1 shows one example of clinical case published in English in *Archive of Clinical Cases*^9^. We can see that this case first describes the patient involved and the reason of the consultation (complaints of the patient). Then it indicates the examination results and the history of the patient. After the diagnosis is done, the patient undergoes medical procedures. Finally, the issue of the intervention is indicated. As one can see, the clinical part of publications on clinical cases may be very similar to real clinical documents. Nevertheless, misspellings, which are quite frequent in clinical documents, may be less frequent in publications with clinical cases.

### Annotation of the corpus

The purpose of the annotations is to enrich the corpus with morpho-syntactic and semantic information. Annotation at the morpho-syntactic level is performed with a tool developed in-house and freely available as an online web-service at https://allgo.inria.fr/app/tagex. Indeed, we take advantage of the clinical cases corpus to develop a PoS-tagger specific to the biomedical and clinical French texts. The purpose of this PoS-tagger is to improve how biomedical terminology is handled and to take into account idiomatic expressions with specific syntactic roles, such as nominal (*point de vue* (point of view)), adverbial (*tout à fait* (fully, completely), *de temps en temps* (sometimes)) or prepositional (*en faveur de* (in favor of), *en l’absence de* (in absence of) groups. PoS-tagging is done by training a CRF model [39] and adopting an Active Learning framework with a previously proposed strategy [40] to select new examples. The process starts with a small amount of manually annotated data which is used to train a first CRF model; this set is then used to annotate new data, which are partly manually corrected and used to train a second CRF model, etc. In this way, it provides a large set of annotated sentences with minimal human supervision. Lemmatization is done by learning rewriting rules relying on the final substring of the word, the size of the substring, and the PoS tag of the word. The application of the rules depends on the PoS tag and the size of the substring. For instance, for a word with a given PoS tag, if there exists a rule matching its 7 last letters, that rule is applied. If no such rule exists, a rule matching the 6 last letters is searched, etc.

In addition, several layers of semantic annotation, shown in Table 1, are performed automatically.

- *Concept Unique Identifiers (CUI)*. The CUI tagging corresponds to the French terms from the UMLS [41]. This tagging is done for single and multi-word terms. For multi-word terms, the annotation respects the IOB format, like for instance for the two-word term *vitamine B12*:

**Table 1:** Example of the annotated sentence from the corpus (B-u-x stands for the beginning of the uncertainty cue or scope number x, B-n-y for the negation cue or scope number y)

word	PoS	lemma	uncert.	uncert.	CUI	neg	neg
			cue	scope		cue	scope
L’	B-determiner	le	O	O	O	O	O
adolescent	B-common_noun	adolescent	O	O	B-C0205653	O	O
parait	B-present_verb_form	paraître	B-u-1	O	O	O	O
triste	B-adjective	triste	O	B-u-1	O	O	O
et	B-coordination_conjunction	et	O	O	O	O	O
ne	B-adverb	ne	O	O	O	B-n-1	O
parle	B-present_verb_form	parler	O	O	O	O	B_n-1
pas	B-adverb	pas	O	O	O	I-n-1	O
.	B-ending_punctuation_mark	.	O	O	O	O	O

**Table:** 

...	O
vitamine	B-C0042845
B12	I-C0042845
...	O
  In the current version of the corpus, in case of several concurrent CUIs, only the longest, and supposedly more precise, CUIs are kept. For instance, *carence en vitamine B12(deficiency in B12 vitamin)* (C0042847) will be preferred to *vitamine B12* (C0042845);
- *Negation*. Negation indicates whether a given disorder, procedure or treatment is present or not in the medical history and care of a given patient. Therefore, detecting negation in biomedical texts has become one of the unavoidable pre-requisites in many information extraction tasks. As presented in [38], 200 clinical cases (87,487 word occurrences) from the CAS corpus were manually annotated by two annotators. In this subset of the CAS corpus, out of 3,811 sentences, 804 sentences contain at least one instance of negation, which corresponds to 21% of negated sentences. The inter-annotator agreement [42] is 0.90 for negation cues and 0.81 for negation scope. Besides, additional 6,601 sentences from another corpus (corpus ESSAI built with clinical trial protocols) were annotated as well to find 1,025 more negative sentences. Those manually annotated data were then exploited for training several supervised learning models. We first train a CRF model for negation cue detection, and secondly, we train a bidirectional long short-term memory neural network with a CRF prediction layer (BiLSTM-CRF) for negation scope detection. The results presented in [38] go up to 0.97 F-measure for cue detection and 0.91 for scope detection on sentences from the CAS corpus;
- *Uncertainty*. Uncertainty is also an integral part of medical discourse and must be taken into account for a more precise computing of the status of disorders, procedures and treatments. Uncertainty cues correspond to simple and complex lexical cues like *probablement, certainement* (probably, certainly) and morphological cues like conditional verbs *indiquerait, proviendrait* (should indicate, may be caused by). Similarly to negation, 200 clinical cases have been annotated by one human annotator for marking up the uncertainty cues and their scope. Overall, out of 3,811 sentences, 226 sentences contain uncertainty, which corresponds to 6% of uncertain sentences. An additional 6,601 sentences from the ESSAI corpus have also been annotated to find 631 uncertain sentences. Similarly to negation, these annotated data have been used to train several supervised learning models for the detection of uncertainty cues and their scope. Hence, we obtain up to 91.30 F-measure for cue detection and 86.73 for scope detection in sentences from the CAS corpus [38].

Since there may be several markers of negation and uncertainty in a sentence, they are numbered with their scopes accordingly: the scope of each detected cue is processed independently by the model. Table 2 provides an example of this type, with three cues and their scopes in the sentence *Il n’y avait pas d’argument pour une infection pariétale et vasculaire à CMV : absence d’ulcérations cytomégaliques et immunohistochimie négative.* (There were no arguments for the parietal and vascular CMV infection: no cytomegalic ulcerations and negative immunohistochemistry.)

**Table 2:** Example of negation annotation with several negation cues and their corresponding scopes

Il	O	O	vasculaire	O	I-n-1
n’	B-n-1	O	à	O	I-n-1
y	0	B-n-1	CMV	O	I-n-1
avait	O	I-n-1	:	O	O
pas	I-n-1	O	absence	B-n-2	O
d’	O	I-n-1	d’	I-n-2	O
argument	O	I-n-1	ulcérations	O	B-n-2
pour	O	I-n-1	cytomégaliques	O	I-n-2
une	O	I-n-1	et	O	O
infection	O	I-n-1	immunohistochimie	O	B-n-3
pariétale	O	I-n-1	négative	B-n-3	O
et	O	I-n-1	.	O	O

### Comparison with clinical narratives

The purpose of the comparison between clinical cases and clinical narratives is to assess the degree of similarity between the two sources of clinical data. This comparison is performed in two ways:

- 4,268 cases (all medical specialties taken together) are compared with randomly selected 4,268 narratives from various specialities;
- 951 cases related to nephrology are compared with 951 randomly selected narratives with the ICD-10 codes related to nephrology (N00 to N19).

Clinical cases are related to specific clinical situations and describe precise situation (diagnosis, surgery, chemotherapy...) for one patient mainly, but sometimes for more than one patient, and the clinical issue for that patient (improvement, stable, death...). The clinical narratives at our disposal come from Rennes University Hospital. Each narrative is related to a patient’s hospitalization and can contain multiple notes depending on the length of stay and the medical specialties involved. We should keep this specificity in mind, because it may cause some statistical differences between the two corpora.

Before being compared, the documents are split into sentences and words, the words are converted into lowercase and numerical sequences are removed. Thus, we expect to better capture the lexical similarity and variety of the two corpora.

## Results

In this section, we first describe the content of the CAS corpus (“Content of the corpus”), then the annotation output and statistics (“Annotation of the corpus” sections).

### Content of the corpus

Currently, the corpus contains 4,900 clinical cases in French, totaling nearly 1.7M word occurrences. This gives 350 word occurrences per clinical case on average. This corpus is continuously updated, as we are periodically adding new, non-annotated clinical cases. In the next section, we present some of the annotations performed on the version of this corpus containing 4,268 clinical cases (over 1.5M word occurrences).

### Annotation of the corpus

The corpus contains several layers of morpho-syntactic and semantic annotations. The tags follow the IOB (Inside-Outside-Begin) format.

In Table 1, the second and third columns give the PoS-tagging and lemmatization for the sentence *L’adolescent paraît triste et ne parle pas* (The teenager seems to be sad and does not speak). As the Table shows, we have chosen to use explicit PoS-tags (*determiner, common_noun, adjective*, etc.). When a given tag corresponds to one word, the tag is prefixed with *B* for *Beginning* position. In the next columns, the words receive the CUI, negation and uncertainty annotations, when relevant, as well as the scope of negation and uncertainty.

Table 3 summarizes the statistics of the corpus. We indicate the size of the corpus (number of clinical cases and words). When the clinical cases are issued from scientific publications, they are accompanied by their discussions. 2,626 such discussions have been collected together with the 4,268 cases. They contain over 1.8M word occurrences. We also indicate the average number of units automatically recognized in clinical cases for each category (CUIs, and uncertainty and negation cues).

**Table 3:** Statistics on the CAS corpus: current size and annotations (average number by sentence)

*type*	*nb.*
clinical cases	4,268
word occurrences	1.5M
discussions	2,626
word occurrences	1.8M
CUI	2.34 / sent.
uncertainty	0.23 / sent.
negation	0.22 /sent.

The PoS-tagger has been evaluated on a 3,000 token excerpt from the CAS corpus and compared to TreeTagger [43], a commonly used PoS-tagger. Three human annotators independently annotated the tagger output in terms of errors of sentence segmenting and tokenization, errors of PoS and errors of lemmas. The annotation errors were then discussed to reach a consensus. Table 4 presents the results. We can see that our tagger seems to be more suitable for the processing of medical texts as it provides results with fewer errors.

**Table 4:** Number of errors for each category (segmenting, PoS, lemmas)

	segmenting	PoS	lemmas
TreeTagger	32	90	83
Our tagger	5	48	37

In order to evaluate the automatic annotation of CUIs, we manually developed the reference annotation on a subset of the corpus concerning the anatomy concepts. For this, two human annotators independently annotated a 30,000-token excerpt of the corpus. Annotations were then discussed to reach a consensual annotation. This reference annotation was then used to evaluate our automatic CUI annotation, considering of course only the CUIs related to the ANATOMY semantic type of the UMLS. Table 5 sums up the performance obtained in terms of precision and recall. Note that most of the anatomy missed by our tool are in fact due to the absence of the corresponding concept in the UMLS (like *cavités pyélo-calicielles* (pyelocaliceal cavities), *parenchyme* (parenchyma), *avant-pieds* (metatarsal bones), or *canal carpien* (carpal tunnel)).

**Table 5:** Performance of our CUI-annotation system on Anatomy concepts in terms of precision and recall

	Nb of tokens	Nb of reference annotation	Precision	Recall
Our system	29,942	511	98%	76.7%

## Discussion

In this Section, we first discuss the comparison of the CAS corpus with similar clinical corpus (clinical narratives from Rennes University Hospital (“Comparison of the CAS corpus with similar clinical narratives”)) and then discuss the utility of this kind of corpus (“Utility of the corpus” sections).

### Comparison of the CAS corpus with similar clinical narratives

Table 6 provides with a global comparison between clinical cases and clinical narratives. For the same number of documents and patients (4,268 and 951), it indicates the number of sentences, the number of words (occurrences and types), the average number of sentences per document, and the average number of words per sentence. One can see that narratives are longer than clinical cases in terms of sentences, and word occurrences and types. As we explained, this is mainly due to the fact that each medical specialty involved produces at least one note. Depending on the length of stay, many notes may be added to the patient’s record. Hence, we observe a greater variance of vocabulary and a higher number of sentences in clinical narratives. Another difference, which may be due to the source of the corpora, is that the average number of words per sentence is higher in clinical cases. There are three reasons for this:

- clinical narratives may indeed contain table-like presentation of examinations, prescriptions and lab results, while in clinical cases this information is usually presented as part of sentences;

**Table 6:** Comparison between clinical cases and clinical narratives; all specialities (upper part), nephrology (lower part)

*type*	*clinical narratives*	*clinical cases*
nb of patients	4,268	4,268
nb of sentences	633,786	82,144
nb of words (occurrences)	5,076,648	1,704,940
nb of unique words (types)	51,369	46,629
avg nb of sentences/document	148.50	19.25
avg nb of words/sentence	8.01	20.76
nb of patients	951	951
nb of sentences	195,036	24,271
nb of words (occurrences)	1,613,245	502,636
nb of unique words (types)	28,529	23,733
avg nb of sentences/document	205.09	25.52
avg nb of words/sentence	8.27	20.71
- because clinical cases are part of scientific articles, the sentences they contain are usually well formed and provide exhaustive information about a given issue on patients;
- clinical narratives contain many administrative information (address, dates, department contacts, politeness expressions, etc.) which are not present in clinical cases.

In Table 7, we indicate the textual similarity between the two corpora compared. Here again, the values are provided for the same number of documents and patients (4,268 and 951). The similarity is computed with the Jaccard index [44]. This index is a statistic measure used for evaluating the similarity and diversity of sample sets. When applied to textual data, it evaluates the textual and lexical similarity of the corpora compared. The index is defined as the size of the intersection divided by the size of the union of the corpora: $J_{A,B}=\frac {A \cap B}{A + B - (A \cap B)}$, where *A* and *B* are the two corpora compared. The values of the Jaccard index are between 0 (no intersection) and 1 (same content). Hence, the closer the value to 1 the higher the similarity of the two corpora. As can be seen in Table 7, the similarity values are high (0.6943 at lowest) and they increase when the sentence length is increased. The similarity values are higher on the larger corpus (4,268 documents) than on the nephrology subset (951 documents) when all the sentences are considered. They are very high (up to 0.9727) on longer sentences. This indicates that the two corpora have lexically similar contents which may be comparable on larger samples of whole documents. This is a positive issue of the comparison between these two corpora. From our point of view, it may indicate that resources, methods and tools developed on the CAS corpus can be effectively exploited on real clinical narratives created in hospitals.

**Table 7:** Similarity between clinical cases and clinical narratives; all specialities (upper part), nephrology (lower part)

*type*	*value*
4,268 patients	
Jaccard (whole documents)	0.7054
Jaccard (sentences with at least 5 words)	0.6973
Jaccard (sentences with at least 10 words)	0.6979
Jaccard (sentences with at least 20 words)	0.8663
Jaccard (sentences with at least 30 words)	0.9715
951 patients	
Jaccard (whole documents)	0.6943
Jaccard (sentences with at least 5 words)	0.6841
Jaccard (sentences with at least 10 words)	0.6826
Jaccard (sentences with at least 20 words)	0.8722
Jaccard (sentences with at least 30 words)	0.9727

We also computed the out of vocabulary (OOV) words in clinical cases. The purpose is to discover what the specificity of the vocabulary is, whether there are misspellings and what the types of those misspellings are. The comparison is done with *Lexique380*,^10^ a lexicon created by psycholinguists. It contains over 135,000 entries, among which inflections for nearly 35,000 lemmas of nouns, adjectives and verbs. This lexicon can be considered as reference lexicon representing the average language performance in French. Among the OOV words (i.e. out of *Lexique380*), we can find technical medical terms, sequences with segmentation problems (*léquipe* (the team), *dopplerhépatique* (hepatic doppler) or *maines* from *humaines* (human)), missing or excessive diacritics (*eruption* (eruption), *realisés* (done), *brulure* (burn), *révèlé* (shown), *acidóse* (acidosis), *téte* (head)), and other misspellings (*rhytme* (rythm), *diabèt* (diabetes), *cliniquemen* (clinically), *agricultureil* (agricultural), *infarctuse* (infarction), *éxtrimité* (extremety)). The misspellings we find in the clinical cases corpus are comparable with the existing typologies [45, 46] and fall into the proposed types: insertion, omission, substitution, transposition, multiple/mixed. Yet, a more exhaustive analysis and comparison of misspellings and their prevalence in French clinical narratives and clinical cases has yet to be done.

### Utility of the corpus

We assume that this kind of corpus is useful for the development and test of automatic tools dedicated for the processing of clinical and medical documents. In addition, the fact that this corpus is freely available for research, will help the sustainability of automatic tools and reproducibility of their results. It may also encourage the competition and robustness of the proposed tools and methods. For instance, the CAS corpus has been used in the French NLP challenges DEFT 2019^11^ and DEFT 2020.^12^ Specific manual annotations have been prepared for this challenge (gender and age of patients, consultation reasons, healthcare outcome in 2019, procedures, signs and symptoms, anatomy, medication and other fine-grained information in 2020). Several teams have been attracted by the challenge and participated on tasks dedicated to information retrieval and extraction. This corpus may be exploited in other NLP challenges.

As it becomes more difficult to access clinical documents, corpora with published clinical cases, which are freely available online, may help researchers to work on this type of data. Let’s also mention the existence of a similar corpus with clinical cases in German [47], whose purpose is also to help working on clinical and medical texts.

## Conclusion

We presented a new corpus in French, called the CAS corpus, which provides medical data close to those produced in the clinical context: description of clinical cases of real or fake patients and their discussion. Overall, the corpus currently contains 4,900 clinical cases, totaling nearly 1.7M word occurrences. A subset of this corpus is currently annotated with several layers of information: morpho-syntactic (PoS-tagging, lemmas) and semantic (the UMLS concepts, uncertainty, negation and their scopes). The corpus is being enriched with more clinical cases published. Besides, other annotation layers are being added and their correctness cross-validated by human annotators.

This corpus has been compared with similar data from Rennes University Hospital. Our analysis showed that there is a strong lexical similarity between these two corpora, which increases when longer sentences are considered. An analysis of out of vocabulary words seems to indicate that misspellings are similar to those proposed in the existing typologies.

The very purpose of our work is to create annotated corpora with clinical cases in French and to make them freely available for research. We expect that this may encourage the development of robust NLP tools for medical narrative documents.

## Abbreviations

Bi-LSTM: Bidirectional Long Short-Term Memory, recurrent neural network, deep learning algorithm
CépiDc: Centre d’épidémiologie sur les causes médicales de Décès – French Epidemiology Department on medical causes of death
CISMEF: Catalogue et Index des Sites Médicaux de langue
Française – French Index of medical websites; CLEF-eHEALTH: international challenge on health-related information retrieval
CRF: Conditional Random Field, machine learning algorithm
CUI: Concept Unique Identifiers for terms from the UMLSv DEFT: Défi Fouille de Textes, NLP challenge on French documents and data
eHealth-KD: NLP challenge for knowledge discovery (health entities and relations)
HAL: open source archive with publications from French researchers
HON: Health On the Net, a non for profit organisation which promotes transparent and reliable health information online
i2b2: Informatics for Integrating Biology and the Bedside, an NLP challenge
ICD-10: International Classification of Diseases, 10th revision, used for the identification of health trends and statistics globally and the international standard for reporting diseases and health conditions
IOB: Inside-Outside-Begin format for annotations
ISTEX: French plateform offering to researchers online access to collections with scientific literature from different domains
MIMIC: Medical Information Mart for Intensive Care, corpus with clinical data
n2c2: National NLP Clinical Challenges, NLP challenge
NLM: National Library of Medicine
NLP: Natural Language Processing
OOV: out of vocabulary words
PoS-tagging: part of speech tagging
UMLS: the Unified Medical Language System

## Footnotes

1 https://www.ncbi.nlm.nih.gov/pubmed

2 https://hal.archives-ouvertes.fr/

3 https://www.istex.fr/

4 https://www.i2b2.org/NLP/DataSets/Main.php

5 https://n2c2.dbmi.hms.harvard.edu/

6 https://sites.google.com/site/shareclefehealth/

7 http://www.cepidc.inserm.fr/

8 https://knowledge-learning.github.io/ehealthkd-2019

9 http://www.clinicalcases.eu

10 http://www.lexique.org/

11 https://deft.limsi.fr/2019/

12 https://deft.limsi.fr/2020/
